# Incidence and clinicopathological analysis of portal vein and inferior vena cava thrombosis in autopsy cases of autosomal dominant polycystic kidney disease

**DOI:** 10.1007/s10157-026-02825-w

**Published:** 2026-02-06

**Authors:** Anna Shinozaki, Tomoko Yamamoto, Atsushi Kurata

**Affiliations:** 1 Tokyo Women’s Medical University, Tokyo, Japan; 2 Department of Pathology, Tokyo Women’s Medical University, 8-1 Kawada-Cho, Shinjuku-Ku, Tokyo, 162-8666 Japan

**Keywords:** Autopsy, Autosomal dominant polycystic kidney disease, Inferior vena cava, Portal vein, Thrombus

## Abstract

**Background:**

With regard to complications of portal vein (PV) and inferior vena cava (IVC) thrombosis in autosomal dominant polycystic kidney disease (ADPKD), several cases have been reported based on imaging findings. However, only one autopsy case has been described and no systematic analysis has been conducted to date. This retrospective study aimed to review autopsy cases from our department over the past 37 years to clarify the frequency and background factors of thrombosis formation in ADPKD.

**Methods:**

Among 4001 autopsies performed at our institution from 1987 to 2023, 10 ADPKD cases were identified. We examined the presence of thrombus in these 10 cases and compared pleural effusion and ascites volumes, major organ weights, and clinicopathological factors between cases with thrombus and those without.

**Results:**

Among 10 ADPKD cases, thrombi were identified in four cases in which autopsies were performed relatively recently. These thrombi were distributed in the PV, IVC, and their branches. Compared to non-thrombotic cases, those with thrombi showed a statistically significant increase in kidney weight and tended to have a higher frequency of complications such as sepsis and severe aortic atherosclerosis.

**Conclusion:**

This study reports the first systematic autopsy-based investigation of PV and IVC thrombosis in ADPKD. Thrombosis was found at a high frequency of 40% and appears to have increased in recent years. Increased kidney weight was associated with thrombosis formation, and blood stasis due to compression by enlarged kidneys is considered the primary cause. Further case accumulation and elucidation of the pathophysiology involved are anticipated.

## Introduction

Autosomal dominant polycystic kidney disease (ADPKD) is an autosomal dominant hereditary disorder characterized by multiple, progressive cyst formations in both kidneys. It is caused by mutations in the *PKD1* gene on the short arm of chromosome 16 or the *PKD2* gene on the long arm of chromosome 4 [1, 2]. Mutations in *PKD1* account for approximately 80% of ADPKD cases, while mutations in *PKD2* account for 15% of such cases [2, 3]. Mutations in these genes, which encode proteins localized to the cilia of renal tubular epithelial cells, inhibit intracellular calcium signaling. This leads to uncontrolled cell proliferation, the formation of fluid-filled cysts, distortion of the renal parenchyma, and, ultimately, loss of renal function [4]. The prevalence of ADPKD is approximately 0.1–0.2% in a normal population. In patients aged 35 years or older, hepatic cysts are observed in more than 90% of cases [5]. Other complications include hypertension, nephrolithiasis, cyst infections, cerebral aneurysms, abdominal hernias, and valvular heart disease [2, 5].

In this disease, based on imaging findings, several cases of hepatic venous outflow obstruction, presumably due to hepatic vein (HV) thrombosis, have been reported and one case of portal vein (PV) thrombosis has also been described [6]. However, to the best of our knowledge, no English-language reports on HV and/or PV thrombosis based on autopsy specimens exist, except for one case of HV thrombosis [7]. English-language papers reporting cases of PV or HV thrombosis, to date, are shown in Table 1 [6–14]. As most of these are single case reports, the frequency of thrombosis in the PV and/or HV, including the inferior vena cava (IVC), in ADPKD has not been reported to date. Therefore, this retrospective study aimed to clarify the frequency of PV and/or HV/IVC thrombosis in ADPKD and the reasons for thrombosis formation through a review of autopsy cases at our institution over the past 37 years.

**Table 1:** Cases with PV or IVC/HV thrombosis in ADPKD reported in the English literature

	Imaging diagnosis		Autopsy diagnosis	
PV thrombosis	Tsuchida et al. 2023 [6]	1 case	None	
IVC/HV thrombosis	Torres et al. [8]	4/10 cases*	Tamburrini et al. [7]	1 case
	Perces et al. [9]	1 case		
	Iguchi et al. [10]	1 case		
	Maeda et al. [11]	1 case		
	Murthuraman et al. [12]	1 case		
	Xie et al. [13]	1 case		
	Chan et al. [14]	1 case		

ADPKD: Autosomal-dominant polycystic kidney disease, HV: Hepatic vein, IVC: Inferior vena cava, PV: Portal vein

^*^ Thrombosis was present in 4 out of 10 cases of hepatic venous outflow obstruction

### Materials and methods

This study was approved by the Ethics Committee of Tokyo Women's Medical University (No. 2024–0125). All autopsy records from our Department of Pathology over the 37-year period from 1987 to 2023 were retrieved, and cases clinically diagnosed with ADPKD during their lifetime were extracted. These cases were re-examined to identify the presence of PV, HV, IVC, or other venous thrombosis. In addition, we examined the presence of background clinical factors, such as age, sex, sepsis, history of dialysis, medication with anticoagulant therapy, and cerebral aneurysms. We also examined background pathological factors including cause of death, pleural effusion and ascites volumes, liver, kidney, and spleen weights, hepatic cysts, sepsis findings, and degree of aortic atherosclerosis. All weights and volumes were accurately measured during each autopsy.

Statistical analysis of searched results was performed using a Mann–Whitney *U* test for differences in numerical values between thrombosis complication and non-thrombosis groups. Chi-square tests for differences in the presence or absence of events were performed using EZR statistical software (Saitama Medical Center, Jichi Medical University, Saitama, Japan, Version 1.68). Regarding kidney weight, since some cases involved unilateral nephrectomy, the remaining unilateral kidney weight was used for such cases. Furthermore, first, for cases with both kidneys, the combined weight of both kidneys was used. Second, for cases with both kidneys, the heavier kidney weight was used for comparison. Third, for cases with both kidneys, the average weight of the left and right kidneys was used for comparison.

Furthermore, in cases with thrombus complications, a detailed histopathological re-examination, including thrombus distribution, maximum diameter, and the presence of thrombus organization, was performed to investigate the mechanisms of thrombus formation in ADPKD.

## Results

Of 4001 autopsies performed in our pathology department from 1987 to 2023, 10 cases (0.25%) were clinically diagnosed with ADPKD during their lifetimes (Table 2). The age range was 49 to 87 years, with a male-to-female ratio of 7:3. Four cases showed complicated thrombus formation (Cases 1–4), which, when viewed chronologically by autopsy year, were relatively recent. Two more recent cases without thrombus formation both involved patients receiving anticoagulant therapy for either cerebral infarction or transcatheter aortic valve implantation following aortic valve stenosis.

**Table 2:** Clinical characteristics of ADPKD cases

Case	CO	Age	Sex	Thrombus	Therapy	Cause of death	Other lesions
1	5	60	M	IVC, PV (rt)	Hemodialysis	Sepsis, peritonitis, perforation of colonic ulcer	Cardiac hypertrophy, brain infarction
2	6	59	M	IVC	Hemodialysis	Sepsis, pneumonia	Cardiac hypertrophy, cardiac and brain infarction
3	7	65	M	IVC/HV, PV	Hemodialysis	Multi-organ failure	
4	8	60	F	PV	Hemodialysis Renal transplantation	Sepsis, multi-organ failure	Brain aneurysm
5	1	49	F	–	Cystjejunostomy and fenestration of liver cyst, hemodialysis	Liver abscess	Cardiac hypertrophy, esophageal varix
6	2	87	M	–	Unable to confirm	Hemorrhagic shock, intestinal hemorrhage	
7	3	69	F	–	Unable to confirm	Perforation of colon	Cardiac hypertrophy, cardiac and brain infarction
8	4	71	M	–	Hemodialysis	Brain hemorrhage	Cardiac hypertrophy
9	9	72	M	–	Hemodialysis, renal transplantation	Sepsis, aspergillosis	Cardiac hypertrophy, brain infarction
10	10	66	M	–	Hemodialysis	Sepsis, cardiac tamponade, infection of pacemaker lead	Aortic valve stenosis cardiac hypertrophy, esophageal varix

ADPKD: Autosomal-dominant polycystic kidney disease, CO: Chronological order, F: Female, HV: Hepatic vein, IVC: Inferior vena cava, M: Male, PV: Portal vein, RT: Right

**Fig. 1:**
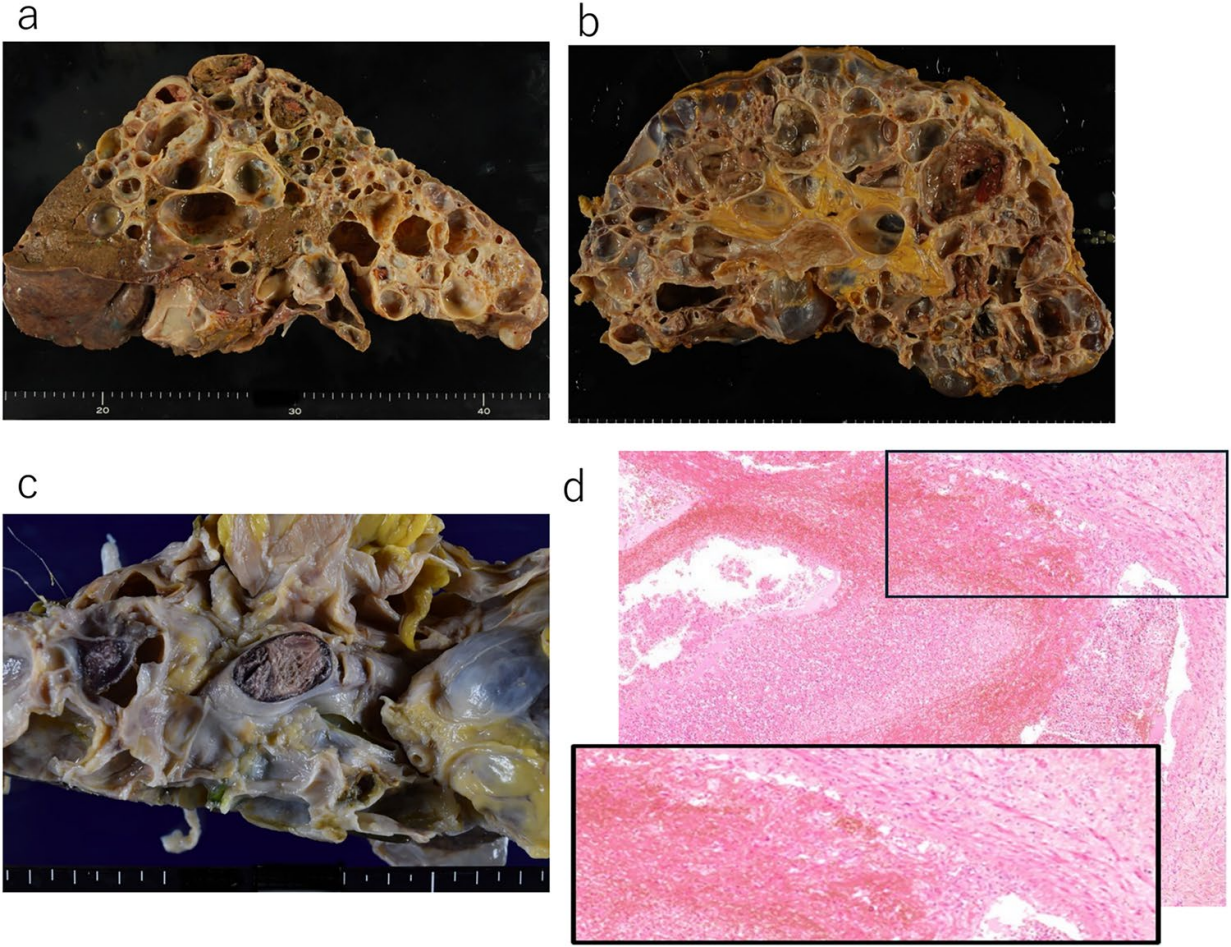
Representative gross and histological findings of organs and thrombi in autosomal-dominant polycystic kidney disease autopsies **a**, **b** Macroscopic appearance of a polycystic liver **a** and kidney **b** observed in Case 3 **c** Macroscopic appearance of a polycystic liver with thrombus formation in the portal vein trunk observed in Case 4 (Table 2). **d** Histological view of a right femoral vein thrombosis observed in Case 2 (40 X). The inset (100 X) shows thrombus organization. Hematoxylin and eosin staining

**Fig. 2:**
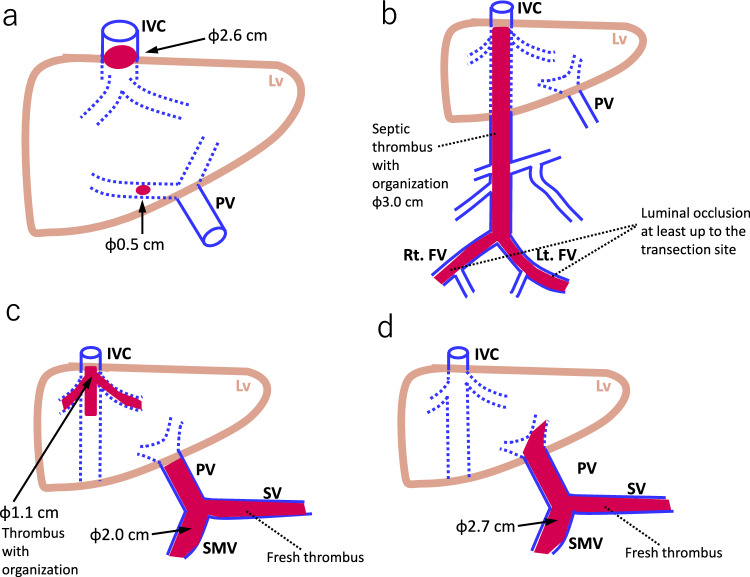
Distribution of thrombi in autopsy cases of autosomal dominant polycystic kidney disease with thrombus complications **a** Case 1: small thrombi (0.5 cm and 2.6 cm in diameter) in the right portal vein (PV) and inferior vena cava (IVC). **b** Case 2: infectious thrombus with organization extending from both femoral veins (FVs) to the IVC. The FV thrombus extends to the intra-abdominal dissection surface. **c** Case 3: a thrombus (1.1 cm) extending from the hepatic vein (HV) to IVC, and a fresh thrombus (2.0 cm) extending from the superior mesenteric vein (SMV) and splenic vein (SV) to the PV trunk. **d** Case 4: a fresh thrombus (2.7 cm) extending from the SMV and SV to the PV trunk Lt: left, Lv: liver, Rt: right

All 10 cases of ADPKD showed multiple cysts in the kidneys and liver. Representative macroscopic sections of the kidney and liver from Case 3 are shown (Fig. 1a, b). In cases with thrombosis, thrombi were observed in the PV and/or IVC and their branches. The breakdown included a total of four cases with overlapping thrombosis in the PV (three cases) and IVC (three cases), with maximum thrombus diameters ranging from 0.5 to 3 cm. Representative macroscopic images of a PV trunk thrombus from Case 4 are shown (Fig. 1c). The following distributions and characteristics were confirmed from autopsy records and histological findings. In Case 1, thrombi formed in the main trunk of the IVC and right PV branch (Fig. 2a). In Case 2, a thrombus with neutrophilic infiltration extended continuously from the IVC to the bilateral femoral veins; this was interpreted as an infectious thrombus. Lumen obstruction, macroscopically evident strong adhesion to the vessel wall, and histologically fibrotic obliteration were observed in many areas (Fig. 2b, Fig. 1d). In Case 3, thrombi were distributed in the IVC, both HVs, and from the PV trunk to the splenic and superior mesenteric veins (SMV), with thrombus organization noted in the distal right HV (Fig. 2c). In Case 4, thrombi were observed from the PV trunk to its left branch, as well as in the splenic and SMV. Thrombus organization was noted in the left branch of the PV (Fig. 2d).

**Fig. 3:**
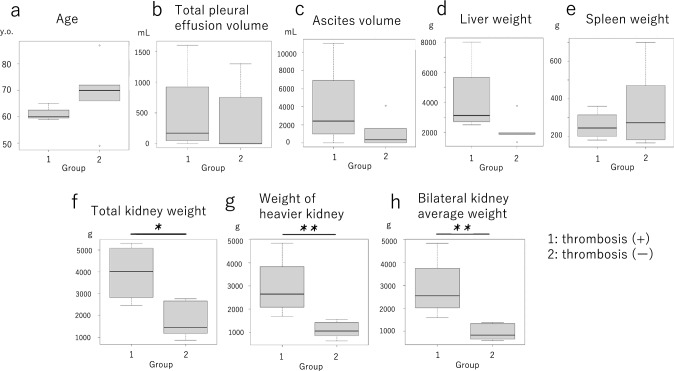
Intergroup comparisons between thrombosis complication (1) and non-thrombosis (2) groups **a**–**e** Comparisons of age, pleural effusion and ascites volumes, and liver and spleen weights, respectively. Medians with interquartile ranges were compared using a Mann–Whitney *U* test, but no significant differences between the two groups were observed for all variables examined. **f**–**h** Comparisons of kidney weights. For single-kidney cases, the weight of that kidney was used. For double-kidney cases, three analyses were performed: using total kidney weight, the heavier kidney alone, and the average weight of both kidneys. For all analytical methods, after comparing medians with interquartile ranges, weight was significantly heavier in the thrombosis complication group according to a Mann–Whitney *U* test (∗ *P* < 0.05; ∗  ∗ *P* < 0.01)

Next, we performed intergroup comparisons of numerical values for age, pleural effusion and ascites volumes, and liver, kidney, and spleen weights between thrombosis complication and non-thrombosis groups (Table 3). It was revealed that ascites volume and liver weight tended to be higher in the thrombosis complication group; however, no significant differences were observed for any item (Fig. 3a–e). Regarding kidney weight, for single-kidney cases, the weight of that kidney was used. For patients with both kidneys present, whether using the combined weight of both kidneys, the weight of the heavier kidney, or the average weight of both kidneys, the thrombosis complication group showed significantly heavier kidney weight than the non-thrombosis group in all analyses (Fig. 3f–h). Thus, kidney weight remained significantly different across all three analytical approaches. The five cases with clinically diagnosed sepsis also showed pathological findings consistent with sepsis, including neutrophilic infiltration of the spleen, immunohistochemically myeloperoxidase-positive cell infiltration into hepatic sinusoids, and a left shift of hematopoietic cells in the bone marrow. Furthermore, based on clinical and pathological findings, the presence or absence of dialysis, sepsis, and severe aortic sclerosis was compared between thrombosis-positive and thrombosis-negative groups. Although no significant differences were found by Chi-square test, a tendency toward higher rates of sepsis and severe aortic atherosclerosis was observed in the thrombosis complication group (Table 4). These results indicate that compared to the non-thrombosis group, the thrombosis group showed significantly increased kidney weight, a tendency toward higher ascites volume and liver weight, and a tendency toward a higher incidence of sepsis and atherosclerosis-related lesions.

**Table 3:** Pathological characteristics of ADPKD cases

Case	CO	Age	Sex	Thrombus	Pleural effusion (rt, mL)	Pleural effusion (lt, mL)	Ascites (mL)	Kidney (rt, g)	Kidney (lt, g)	Kidney (total, g)	Liver* (g)	Spleen (g)	Severe aortic atherosclerosis
1	5	60	M	IVC, PV (rt)	200	40	2000	Resected	2458	2458	2502	270	+
2	6	59	M	IVC	0	0	0	2840	2460	5300	2960	360	+
3	7	65	M	IVC/HV, PV	1000	600	2800	Resected	4840	4840	3300	220	+
4	8	60	F	PV	100	A little	11,000	1500	1700	3200	8000	180	−
5	1	49	F	–	ND	ND	1600	630	540	1170	ND	470	−
6	2	87	M	–	0	0	0	1090	515	1605	1360	280	−
7	3	69	F	–	0	0	0	860	Resected	860	1987	264	+
8	4	71	M	–	0	0	60	1420	1335	2755	1844	165	−
9	9	72	M	–	300	1000	4100	1042	265	1307	1899	183	−
10	10	66	M	–	400	350	600	1560	1100	2660	3770	700	−

ADPKD: Autosomal-dominant polycystic kidney disease, CO: Chronological order, F: Female, HV: Hepatic vein, IVC: Inferior vena cava, lt: Left, M: Male, ND: Not determined, PV: Portal vein, rt: Right

^*^All cases had multiple liver cysts

**Table 4:** Association of thrombosis with dialysis, sepsis, and severe atherosclerosis in ADPKD

	Dialysis	Sepsis	Severe atherosclerosis
Thrombosis( +): n = 4	4/4 cases (100%)	3/4 cases (75%)	3/4 cases (75%)
Thrombosis(-): n = 6	4/6 cases (67%)*	2/6 cases (33%)	1/6 cases (17%)

ADPKD: Autosomal-dominant polycystic kidney disease

^*^In two cases, it was not possible to confirm whether dialysis was performed

## Discussion

This study represents the first investigation, to our knowledge, of the incidence of thrombus complications in autopsy cases of ADPKD. While such autopsy cases may have represented only those with advanced disease, the study design also enabled the direct pathological confirmation of thrombosis, a key strength of our analysis. In this investigation, thrombus complications, such as those in the PV or IVC, occurred in 40% of ADPKD autopsy cases. The breakdown included a total of four cases, comprising three cases of PV thrombosis and three cases of IVC thrombosis (with overlap). This included an autopsy case of PV thrombosis, which, to our knowledge, is the first case report written in Japanese [15], and a second autopsy case of IVC thrombosis. Blood flow stasis in both the PV and IVC seems to be an important factor. This may be attributed to their location adjacent to the kidneys and liver, making these veins susceptible to the effects of any compression of these organs. Therefore, analyzing the PV and IVC together is considered meaningful.

We analyzed the association of thrombosis in ADPKD with various factors. Statistical analysis of 10 ADPKD cases grouped by the presence or absence of thrombotic complications indicated that increased kidney weight is associated with thrombosis. This finding aligns with reports suggesting that in ADPKD, the enlargement of the kidneys compresses the IVC, leading to blood stasis that may promote thrombosis [14]. Regarding PV, it has been pointed out as a general principle, not limited to ADPKD, that compression by tumors or enlarged lymph nodes can similarly cause blood stasis and thrombosis [16]. Furthermore, considering that all thrombotic complications identified in this study occurred in recent ADPKD cases, but that the two latest cases did not show thrombosis and were undergoing anticoagulant therapy, it is possible, although highly speculative, that advances in treatment may have improved the prognosis of patients with ADPKD. Consequently, as cysts grew over time, leading to more pronounced kidney enlargement, the incidence of thrombotic complications may have increased in recent years. This may explain why reports of thrombotic complications in ADPKD were scarce in previous autopsy cases.

Polycystic liver disease is present in over 90% of patients with ADPKD aged 35 years or older and is the most common extra-renal manifestation of this disease [5]. Indeed, multiple hepatic cysts were identified in all 10 ADPKD cases identified in this study. The main complications of hepatic cysts include hemorrhage, infection, and rupture [2, 17]. Hepatic cyst infection occurs in 1% of patients, presenting with fever, fatigue, and right upper quadrant pain [2]. Furthermore, an enlarged liver may exert a mass effect on adjacent organs, potentially causing abdominal distension, dyspnea, and gastroesophageal reflux [17]. It may also cause hepatic venous outflow obstruction [8, 14, 18], with reported cases of Budd–Chiari syndrome, portal hypertension, and biliary obstruction related to extrinsic compression [17]. Although it has been pointed out that cyst formation can cause an enlarged liver that compresses the PV, descriptions of thrombosis are lacking [17]. In this study, cases with thrombotic complications tended to show an increased liver weight, but no significant difference was observed based on the presence or absence of thrombosis (Fig. 3d). Therefore, the increase in liver weight itself may simply reflect a time-dependent increase associated with an improved prognosis in ADPKD.

Furthermore, in autopsy cases with thrombotic complications, sepsis and severe aortic atherosclerosis were each observed in three out of four cases, suggesting a high frequency and indicating that these complications may be associated with thrombus formation. Based on these findings and prior research indicating that the so-called “Virchow’s triad” (stasis, endothelial injury, and hypercoagulability) is implicated in thrombosis formation in ADPKD [14, 15], we revisit this idea. As mentioned above, in ADPKD, the primary cause of blood stasis is thought to be compression of vessels due to enlargement of the kidney and, in some cases, the liver, caused by cyst formation [14, 16, 17]. Furthermore, ascites along with hypoalbuminemia resulting from impaired liver function is also thought to contribute to hypercoagulability [15]. Cases complicated by sepsis represent a state of systemic inflammatory response syndrome (SIRS), triggered by the binding of microorganism-derived molecules to Toll-like receptors present on monocytes and vascular endothelial surfaces, thereby inducing a cytokine storm [19]. This SIRS state is thought to contribute to enhancement of the blood coagulation mechanism. Furthermore, infection promotes the expression of tissue factor (factor III) on the vascular endothelial surface, thereby enhancing extrinsic blood coagulation [20]. Case 2 showed an infectious thrombus, suggesting a somewhat different mechanism of disease from those of other cases; however, underlying blood stasis may have accelerated the thrombus formation. In addition, the elevation of procoagulant factors, such as tissue factor associated with the progression of atherosclerosis, may also have contributed to thrombus formation [21].

The following is expected from our findings: first, awareness will grow regarding the risk of PV and IVC thrombosis in ADPKD. While this study was based on autopsy cases, and it is unclear to what extent it reflects overall trends in ADPKD cases, the finding of more PV and IVC thromboses in recent cases suggests an increase in similar cases in the future. Because this study involved autopsy cases and a small sample size, it is anticipated that when the frequency of similar symptoms is observed to increase, a renewed statistical evaluation may reveal further significant differences. Furthermore, if future research clarifies the mechanisms of thrombosis in PV and IVC in ADPKD, it is hoped this will lead to improvements in treatment methods.

This study had several limitations. First, the autopsy cases described herein may have represented only those with advanced disease since all patients with ADPKD treated at our hospital were not examined by autopsy. Second, 10 cases are not sufficient for statistical analyses. Due to the small sample size, the 40% incidence of thrombotic complications found may not accurately reflect the actual frequency in the general ADPKD population.

In conclusion, this study investigated thrombotic events in ADPKD, including, to our knowledge, the first reported case of PV thrombosis and the second reported case of IVC thrombosis in autopsy specimens of ADPKD in the English literature. Thrombosis in the PV and IVC was observed relatively frequently in 40% of the 10 cases studied. A relationship between thrombosis and kidney weight was suggested, with blood flow stasis due to compression by markedly enlarged organs considered a significant factor. Furthermore, since thrombotic complications were more common in recent cases, their incidence is expected to increase in future due to advances in treatment. Further research is anticipated to contribute to improvements in ADPKD treatment.
